# Preterm Birth and Malaria Susceptibility in Offspring of Uninfected Multigravid Women

**DOI:** 10.1001/jamanetworkopen.2025.32179

**Published:** 2025-09-16

**Authors:** Amadou Barry, Lauren Dang, Youssoufa Sidibe, Djibrilla Issiaka, Santara Gaoussou, Zonghui Hu, Yahia Dicko, Almahamoudou Mahamar, Oumar Attaher, Bacary S. Diarra, Sekouba Keita, Alassane Dicko, Patrick E. Duffy, Michal Fried

**Affiliations:** 1 Malaria Research and Training Center, University of Sciences, Techniques and Technologies of Bamako, Bamako, Mali; 2 Biostatistics Research Branch, National Institute of Allergy and Infectious Diseases, National Institutes of Health, Bethesda, Maryland; 3 Laboratory of Malaria Immunology and Vaccinology, Division of Intramural Research, National Institute of Allergy and Infectious Diseases, National Institutes of Health, Bethesda, Maryland

## Abstract

**Importance:**

Pregnancy malaria (PM) is associated with adverse pregnancy outcomes such as stillbirth, early neonatal death, preterm delivery (PTD), and low birthweight. PM also increases the risk of malaria infection in infants. However, it is unknown whether PTD modifies the risk of malaria infection during early childhood.

**Objective:**

To investigate the association of PM and PTD with child susceptibility to malaria infection and disease.

**Design, Setting, and Participants:**

Pregnant women were enrolled between November 23, 2010, and December 9, 2014, into an observational longitudinal cohort study of mother-child pairs in Ouélessébougou, Mali, an area of high seasonal malaria transmission. Follow-up was completed through pregnancy. Children were enrolled at birth and followed up from January 21, 2011, to July 31, 2016, for as long as 5 years with monthly clinical visits during the malaria transmission season and every 2 months during the dry season. Data were analyzed from November 4, 2024, to July 15, 2025.

**Exposure:**

PM and PTD.

**Main Outcomes and Measures:**

Study end points included *Plasmodium falciparum* infection, clinical malaria, and severe malaria infections. Malaria diagnosis and clinical data were collected during scheduled examinations and unscheduled sick visits. Cox proportional hazards models were used to analyze whether hazards of first malaria infection and first clinical malaria infection were associated with PM and PTD. Associations between the incidence rate of parasitemia and risk factors (eg, maternal infection history, PTD) were estimated using negative binomial models. Cox proportional hazards and negative binomial models with an interaction term among PTD, pregnancy malaria, and gravidity were used to evaluate associations within strata of the 3 covariates.

**Results:**

In 1679 children included in adjusted models (848 female [50.5%] and 831 male [49.5%]), 760 (45.3%) were born during the malaria transmission season and 96 (5.7%) were born preterm. Children were followed up for a mean (SD) of 25.8 (16.1) months. PM was associated with an increased hazard of first malaria infection and first clinical malaria infection in children of women of all gravidities, while PTD (vs full-term delivery) was associated with increased hazard of first malaria infection (hazard ratio, 1.76; 95% CI, 1.05-2.95; *P* = .03) in offspring of multigravid women only. Further, the hazard ratio of first parasitemia for preterm compared with full-term offspring was 2.17 (95% CI, 1.25-3.75; *P* = .006) and 3.63 (95% CI, 1.90-5.93; *P* < .001) in offspring of uninfected secundigravida and multigravida women, respectively. The parasitemia infection incidence rate ratio for PTD was 2.74 (95% CI, 1.80-4.18) in offspring of uninfected multigravida women.

**Conclusions and Relevance:**

In this cohort study of young children, the association between PTD and the hazard of malaria varied based on maternal gravidity and maternal infection history during pregnancy. This information could be used to evaluate the health effects of active monitoring of *P falciparum* infection or adherence to malaria chemoprevention in children born preterm.

## Key Points

**Question:**

Is preterm birth associated with an increased hazard of malaria infection during early childhood?

**Findings:**

In this cohort study of 1679 children, preterm (vs full-term) birth was associated with a higher hazard of first malaria infection in offspring of multigravid and secundigravid women who were not infected during pregnancy. Preterm (vs full-term) delivery was also associated with a higher incidence rate of parasitemia in offspring of multigravid women without infection.

**Meaning:**

These findings suggest that children born preterm have a higher risk of malaria infection, despite having healthy mothers with acquired malaria immunity.

## Teaser

This cohort study evaluates the association of pregnancy malaria and preterm delivery with susceptibility to *Plasmodium falciparum* infection and disease during the early childhood of the offspring.

## Introduction

In areas of stable malaria transmission, children younger than 5 years are the group most susceptible to infection with *Plasmodium falciparum* parasites and malaria disease.^1^ In semi-immune adults, asymptomatic low-density infections are common and reflect acquired immunity over years of exposure.^1^ However, women become susceptible to malaria during pregnancy, despite having acquired substantial immunity over years of exposure to the parasite. The unique features of *P falciparum* infection during pregnancy are the sequestration of infected erythrocytes in the placenta that can be followed by accumulation of inflammatory immune cells and gravidity-dependent susceptibility.^2,3^ In endemic areas, primigravid women are the most susceptible, and during successive pregnancies, women acquire specific antibodies to the parasite sequestering in the placenta.^4^ Pregnancy malaria (PM) is associated with increased risk of poor pregnancy outcomes such as stillbirth, early neonatal death, preterm delivery (PTD), and offspring small for gestational age or with low birthweight.^5,6,7,8,9^ In high malaria transmission zones, the risk of adverse pregnancy outcomes associated with PM vary with gravidity: in primigravid women, PM increased the risks of PTD and stillbirth, while in multigravid women, PM increased the risk of early neonatal death.^9^

PTD and low birthweight are risk factors for mortality and morbidity in young children. Globally, in 2019, 5.3 million deaths occurred in children younger than 5 years, of which 2.44 million (46.0%) occurred in neonates primarily due to PTD.^10^ Mortality in children aged 1 to 59 months was primarily associated with infectious disease, including lower respiratory infections, diarrhea, and malaria. Among children aged 1 to 59 months, PTD was reported as the cause of death in 60 000 cases (2.1%).^10^

Several studies^11,12,13,14^ have reported that PM modifies the risk of malaria infection in infants. In a longitudinal study in Tanzania,^11^ PM at delivery was associated with a shorter time to first infection in offspring. Because PM is associated with increased risks of PTD and modifies *P falciparum* infection during infancy, this study evaluated the association of both pregnancy malaria and PTD with susceptibility to *P falciparum* infection and disease during early childhood.

## Methods

### Ethical Approval and Study Procedures

The study was conducted in Ouélessébougou, Mali, an area with high seasonal malaria transmission. The protocol and study procedures were approved by the Institutional Review Board of the National Institute of Allergy and Infectious Diseases at the National Institutes of Health,^15^ and the Ethics Committee of the Faculty of Medicine, Pharmacy and Dentistry at the University of Bamako, Mali. Pregnant females aged 15 to 45 years without chronic or debilitating disease were enrolled into a longitudinal cohort study of mother-child pairs between November 23, 2010, and December 9, 2014.^9^ Study procedures were as previously described.^9^ Briefly, women were seen monthly for scheduled clinical examinations and for unscheduled visits as needed. Blood samples for malaria diagnosis were collected at enrollment, gestational week 30 to 32, delivery, and sick visits. Gestational age at enrollment was determined by ultrasonographic examination in 97.6% of women, the remaining based on last menstrual period or fundal height.

Children were followed up from birth up to 5 years of age between January 21, 2011, and July 31, 2016, including monthly visits during the malaria transmission season, every 2 months during the dry season, and any time the child was sick.^16^ Visits included clinical examination and malaria diagnosis using blood smear microscopy. This study followed the Strengthening the Reporting of Observational Studies in Epidemiology (STROBE) reporting guideline.

### Clinical Definitions

Maternal infection (PM positive) was defined as the presence of parasites in peripheral or placental blood smear sample or the detection of parasite DNA by nested polymerase chain reaction analysis anytime during pregnancy up to and including delivery.^9^ *P falciparum* infection in children was defined by blood smear microscopy. PTD was defined as viable birth before gestational week 37. Clinical malaria was defined as fever (temperature >37.5 °C) and parasites detected by blood smear microscopy. Severe malaria was defined as parasites detected by blood smear microscopy and one of the following clinical presentations: coma (Blantyre score <3); 2 or more convulsions in the past 24 hours; prostration (inability to sit unaided, or in younger infants, inability to move or feed); hemoglobin level of less than 6 g/dL (per protocol; to convert to g/L, multiply by 10); and respiratory distress (hyperventilation with deep breathing, intercostal recessions, and/or irregular breathing).

### Statistical Analysis

**Figure 1.**
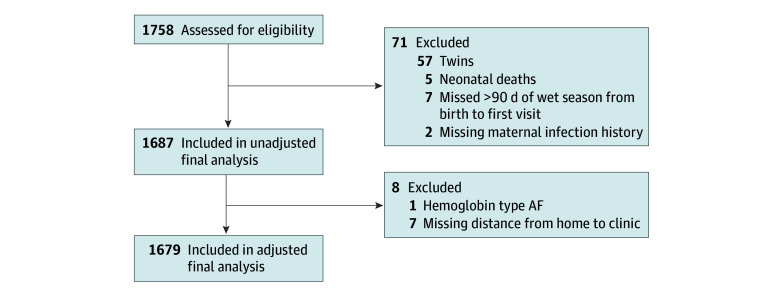
Flow Diagram of Study Population

Data were analyzed from November 4, 2024, to July 15, 2025. The analysis included 1687 children (Figure 1). Missingness was low for all variables except hemoglobin type, which was missing for 164 children. It is not biologically plausible that the other baseline covariates would be associated with hemoglobin type. For this reason, missing hemoglobin types were imputed with a single imputation based on the proportion of nonmissing hemoglobin types in the dataset.

Kaplan-Meier survival analysis was used to estimate the percentage of children without a detected episode of parasitemia for the offspring of mothers with (PM-positive) and without (PM-negative) infection over time, with a log-rank *P* value for the difference in time to first episode of parasitemia curves. Two-sided *P* < .05 indicated statistical significance. Cox proportional hazard models with robust SEs were used to evaluate whether the hazard of first parasitemia was associated with maternal infection history, and separately with PTD. Because we did not expect the proportional hazards assumption to be true, the target parameter estimated by each of these models should be interpreted as a weighted mean of the hazard ratio (HR) over time.^17^ The associations between the incidence rate of parasitemia and risk factors, including maternal infection history and PTD, were estimated using negative binomial models with an offset for the log of follow-up time and robust SEs. We also fit these Cox proportional hazards and negative binomial models with an interaction between PTD and categories of both maternal infection history and gravidity. By taking the sum of relevant coefficients, estimating CIs for these linear combinations of coefficients based on the model covariance matrices, and converting the estimates and CIs to the appropriate scale, we estimated the association between PTD and the hazard of first parasitemia and incidence rate of parasitemia among strata of maternal infection and gravidity. The associations between risk factors and the incidence of severe malaria episodes per person-years at risk for a first episode of severe malaria were estimated by a Poisson model with offset for the log of follow-up time at risk and robust SEs. Selection between Poisson vs negative binomial models for the incidence rate ratio (IRR) estimates was made based on diagnostics, including a nonparametric overdispersion test and visualization of quantile-quantile scaled residual plots.^18^ The association between odds of parasitemia longitudinally throughout the follow-up period and maternal pregnancy infection history was assessed using a generalized estimating equation logistic regression model with a first-order autoregressive working correlation structure and robust SEs. This model was chosen so that the statistical estimates would be comparable to a past study of the association between odds of parasitemia longitudinally during childhood and placental malaria infection.^11^ Odds ratio estimates were converted to relative risks via the method of substitution.^19^ Models were adjusted for gravidity (nonstratified models) and the following variables: hemoglobin type (AA, AC [including 3 CC], or AS [including 3 SS and 6 SC]), maternal insecticide-treated net use, calendar day of birth modeled as a flexible cubic spline with a knot point at the median and distance from home to clinic, parental educational level, and growth rate. Children were censored at the last visit before they missed more than 90 days of follow-up during the malaria transmission season. Because these results are meant to be exploratory, suggesting associations that may be followed up in future work, no adjustment for multiple testing was performed. Analyses used R, version 4.4.0 (R Program for Statistical Computing).

## Results

### Association Between PM and Time to First Parasitemia

We included 1687 mother-child pairs in the analysis after excluding pregnancies resulting in miscarriage, stillbirth, and neonatal death (Figure 1). Mean (SD) maternal age at enrollment was 24.2 (6.1) years. Among these participants, 1128 women (66.9%) experienced at least 1 infection during pregnancy, 96 pregnancies (5.7%) resulted in PTD. Among the 1679 children included in the adjusted analysis (848 female [50.5%] and 831 male [49.5%]), 760 (45.3%) were born during the malaria transmission season (Table 1 and eTable 1 in Supplement 1).

**Table 1.** Study Population

Characteristic	Maternal group by delivery and malaria status											
	Primigravid, PM positive		Primigravid, PM negative		Secundigravid, PM positive		Secundigravid, PM negative		Multigravid, PM positive		Multigravid, PM negative	
	Full-term (n = 245)	PTD (n = 33)	Full-term (n = 93	PTD (n = 11)	Full-term (n = 198)	PTD (n = 17)	Full-term (n = 103)	PTD (n = 6)	Full-term (n = 616)	PTD (n = 19)	Full-term (n = 336)	PTD (n = 10)
Gestational age at enrollment, mean (SD), wk	20.62 (6.47)	23.13 (6.45)^a^	20.98 (7.73)	24.79 (8.76)^a^	20.41 (6.41)	23.42 (7.84)^a^	20.35 (7.48)	26.76 (5.34)^b^	20.59 (6.92)	22.54 (8.18)^a^	21.09 (6.69)	18.19 (8.29)^a^
Birth during transmission season, No. (%)^c^	111 (45.3)	25 (75.8)^b^	40 (43.0)	6 (54.6)^a^	93 (47.0)	11 (64.7)^a^	46 (44.7)	3 (50.0)^a^	307 (49.8)	8 (42.1)^a^	110 (32.7)	5 (50.0)^a^
Duration of child follow-up, mean (SD), mo	21.5 (15.7)	18.72 (15.8)^a^	21.81 (14.5)	24.18 (15.3)^a^	22.01 (15.8)	14.58 (10.7)^a^	20.57 (14.0)	24.24 (15.6)^a^	24.31 (15.7)	17.19 (13.4)^b^	26.11 (16.1)	23.52 (15.1)^a^
Distance to clinic, mean (SD), km	2.94 (4.37)	4.79 (7.99)^a^	2.16 (5.74)	3.09 (7.27)^a^	3.97 (6.50)	5.82 (6.98)^a^	2.42 (4.35)	5.67 (11.92)^a^	5.18 (7.59)	8.53 (11.76)^a^	3.66 (7.56)	3.20 (3.67)^a^
Hemoglobin type, % with mutation (95% CI)^a^												
AC	10.61 (7.05-15.16)	12.12 (3.40-28.20)	9.68 (4.52-17.58)	27.27 (6.02-60.97)	10.10 (6.28-15.17)	11.76 (1.46-36.44)	11.65 (6.17-19.47)	0	8.28 (6.23-10.74)	5.26 (0.13-26.03)	12.20 (8.90-16.19)	30.00 (6.67-65.25)
AS	8.57 (5.38-12.80)	3.03 (0.08-15.76)	8.60 (3.79-16.25)	0	10.10 (6.28-15.17)	23.53 (6.81-49.90)	10.68 (5.45-18.31)	16.67 (0.42-64.12)	10.06 (7.80-12.72)	0	9.23 (6.26-12.84)	10.00 (0.25-44.50)
Maternal ITN use, No. (%)	92 (37.6)	15 (45.5)	45 (48.4)	5 (45.5)	105 (53.0)	10 (58.8)	56 (54.4)	2 (33.3)	359 (58.3)	8 (42.1)	179 (53.3)	7 (70.0)
Severe malaria in child during follow-up, No. (%)^a^	22 (9.0)	2 (6.1)	4 (4.3)	0	19 (9.6)	2 (11.8)	2 (1.9)	0	46 (7.5)	2 (10.5)	24 (7.1)	1 (10.0)

Abbreviations: PM, pregnancy malaria; PTD, preterm delivery; ITN, insecticide-treated net.

^a^ No significant differences in the comparison of PTD with term deliveries.

^b^ *P* < .05 in the comparison of PTD with term deliveries.

^c^ Peak malaria transmission in Ouélessébougou, Mali, occurs during the rainy season from July to December.

PM at delivery has been associated with infant risk of *P falciparum* infection and disease.^11,12,13,14^ Therefore, we first evaluated the association between PM and the child’s susceptibility to malaria infection during early childhood. In estimated cumulative infection risk, by the age of 1 year, 46% of children had *P falciparum* infection, and by 3 years of age, 83% experienced at least 1 infection. Median time to first infection was 49.9 (95% CI, 47.3-54.6) weeks in children of PM-positive mothers and 80.9 (95% CI, 72.4-95.2) weeks in children of PM-negative mothers (eFigure in Supplement 1). In the adjusted model, the instantaneous risk of first *P falciparum* infection was 1.56 (95% CI, 1.34-1.82; *P* < .001) times greater in offspring of PM-positive mothers compared with offspring of PM-negative mothers (eTable 2 in Supplement 1). To evaluate whether gravidity modified this association, HRs were estimated in models stratified by gravidity. Children born to PM-positive mothers of all gravidities were infected earlier compared with children of uninfected mothers (eFigure in Supplement 1). In the Cox proportional hazards regression model, the adjusted HR (AHR) of first malaria infection was significantly higher in children born to PM-positive compared with PM-negative primigravid (1.86; 95% CI, 1.31-2.63; *P* < .001), secundigravid (1.40; 95% CI, 1.00-1.97; *P* = .049), and multigravid (1.54; 95% CI, 1.27-1.87; *P* < .001) women (eTable 2 in Supplement 1). We then assessed the odds ratios and relative risks of becoming infected during infancy to early childhood in relation to PM status (eTable 3 in Supplement 1). Compared with the offspring of PM-negative multigravid women, the adjusted relative risk of *P falciparum* infection longitudinally was increased in children of PM-positive primigravid (1.43; 95% CI, 1.17-1.74), secundigravid (1.37; 95% CI, 1.09-1.72), and multigravid (1.45; 95% CI, 1.22-1.73) women.

### Association of PTD With Susceptibility to Infection

**Figure 2.**
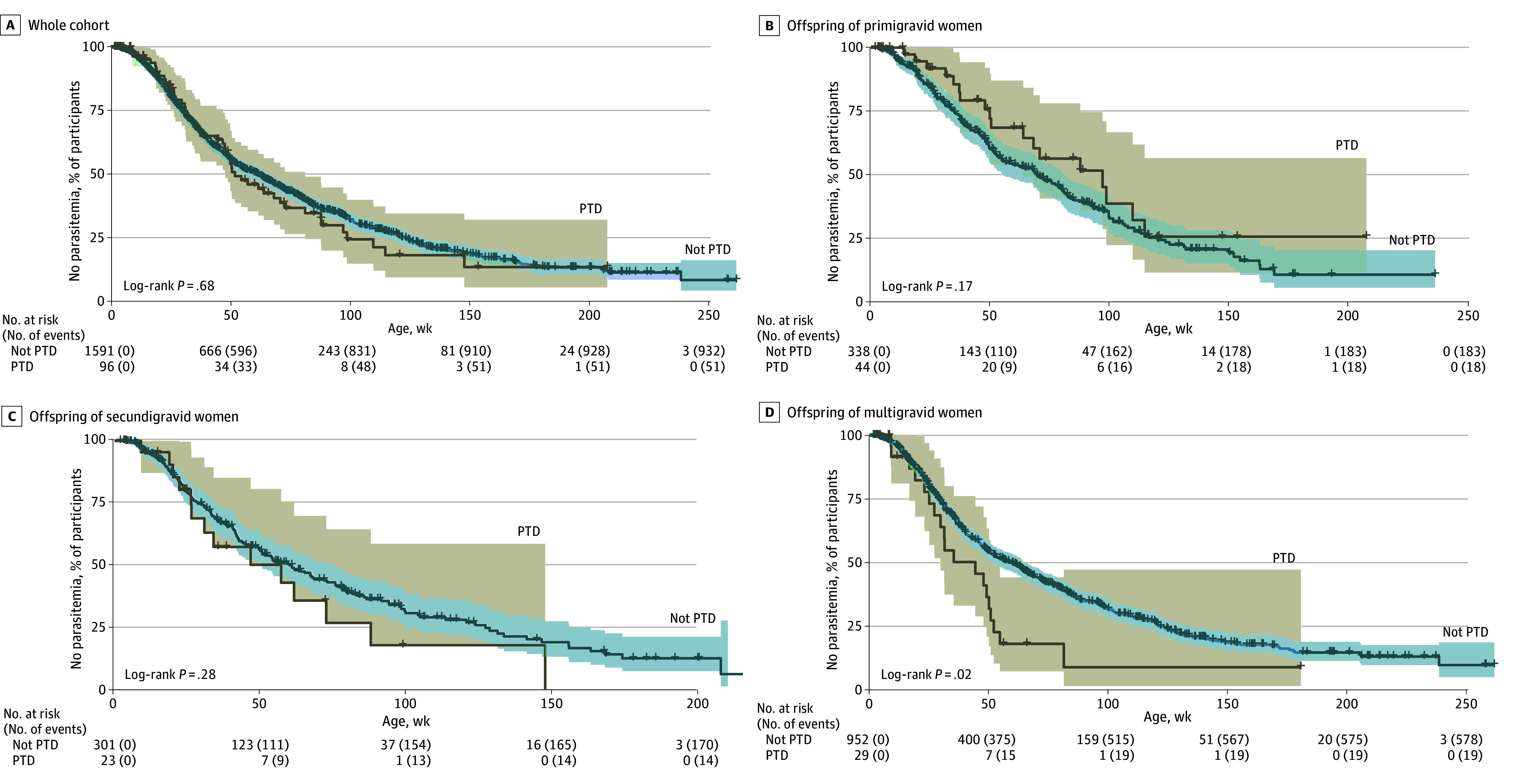
Kaplan-Meier Estimate of Percentage of Children Without Parasitemia Over Time by Preterm Delivery (PTD) Status Data are plotted as unadjusted age to first malaria infection. In adjusted Cox proportional hazards models (Table 2), PTD was associated with a significantly higher hazard of first parasitemia in the offspring of multigravid women.

Age at first malaria infection among children of primigravid, secundigravid, and multigravid women by PTD status is shown in Figure 2. To estimate the HR of first infection for children born preterm compared with those born at full term, a Cox proportional hazards regression model was fitted. In the adjusted model, the hazard of first infection was 1.12 (95% CI, 0.85-1.49) times higher for preterm compared with full-term children; however, this increase was not significant (*P* = .40) (Table 2). In models stratified by gravidity (Table 2), maternal infection was uniformly associated with a higher hazard of first *P falciparum* infection in all children; PTD was associated with a hazard of first infection of 1.76 (95% CI, 1.05-2.95) times the hazard for full-term children among the offspring of multigravid women.

**Table 2.** Unadjusted and Adjusted HR for First Parasitemia and IRR for Parasitemia During Infancy and Early Childhood Among Preterm and Term Newborns^a^

Term	Unadjusted HR (95% CI)	*P* value	Adjusted HR (95% CI)	*P* value	Unadjusted IRR (95% CI)	*P* value	Adjusted IRR (95% CI)	*P* value
**All**								
PTD	1.06 (0.81-1.39)	.70	1.12 (0.85-1.49)	.40	1.04 (0.82-1.34)	.70	1.08 (0.84-1.38)	.60
Maternal *P falciparum*	1.50 (1.31-1.73)	<.001	1.56 (1.34-1.82)	<.001	1.42 (1.24-1.62)	<.001	1.41 (1.24-1.61)	<.001
Gravidity								
Primigravid	0.87 (0.75-1.02)	.08	0.88 (0.74-1.03)	.10	0.84 (0.73-0.97)	.02	0.89 (0.77-1.03)	.10
Secundigravid	1.01 (0.86-1.19)	.90	1.02 (0.86-1.21)	.80	0.89 (0.77-1.04)	.20	0.92 (0.79-1.07)	.30
Multigravid	1 [Reference]	NA	NA	NA	1 [Reference]	NA	NA	NA
ITN use	0.79 (0.69-0.89)	<.001	0.81 (0.70-0.93)	.002	0.85 (0.76-0.95)	.005	0.94 (0.83-1.05)	.20
Hemoglobin AC level	1.01 (0.82-1.26)	.90	0.89 (0.70-1.15)	.40	0.91 (0.77-1.09)	.30	0.95 (0.79-1.13)	.50
Hemoglobin AS level	0.81 (0.66-0.99)	.049	0.79 (0.64-0.98)	.03	0.87 (0.71-1.06)	.20	0.87 (0.71-1.07)	.20
Distance to clinic	1.04 (1.03-1.06)	<.001	1.04 (1.03-1.05)	<.001	1.05 (1.04-1.06)	<.001	1.04 (1.04-1.05)	<.001
**Primigravid**								
PTD	0.71 (0.45-1.136)	.20	0.66 (0.39-1.11)	.10	0.71 (0.44-1.13)	.10	0.71 (0.43-1.16)	.20
Maternal *P falciparum*	1.95 (1.41-2.69)	<.001	1.87 (1.32-2.66)	<.001	1.81 (1.29-2.53)	.001	1.72 (1.24-2.40)	.001
ITN use	0.80 (0.61-1.06)	.10	0.95 (0.70-1.27)	.70	0.84 (0.65-1.07)	.20	0.97 (0.75-1.26)	.80
Hemoglobin AC level	1.06 (0.68-1.65)	.80	0.94 (0.57-1.56)	.80	0.96 (0.66-1.41)	.80	1.05 (0.68-1.62)	.80
Hemoglobin AS level	0.69 (0.38-1.27)	.20	0.70 (0.39-1.25)	.20	0.83 (0.50-1.37)	.50	0.84 (0.49-1.42)	.50
Distance to clinic	1.06 (1.03-1.10)	<.001	1.09 (1.03-1.09)	<.001	1.07 (1.04-1.09)	<.001	1.06 (1.04-1.09)	<.001
**Secundigravid**								
PTD	1.35 (0.84-2.18)	.20	1.31 (0.86-1.99)	.20	1.11 (0.68-1.81)	.70	0.93 (0.61-1.42)	.70
Maternal *P falciparum*	1.35 (0.98-1.85)	.07	1.40 (0.99-1.96)	.05	1.38 (0.99-1.91)	.06	1.34 (0.998-1.81)	.05
ITN use	0.88 (0.66-1.18)	.40	0.82 (0.60-1.11)	.20	1.04 (0.79-1.36)	.80	1.08 (0.83-1.40)	.60
Hemoglobin AC level	0.54 (0.28-1.04)	.07	0.53 (0.28-0.96)	.048	0.42 (0.25-0.70)	.001	0.41 (0.24-0.70)	.001
Hemoglobin AS level	0.57 (0.33-0.96)	.04	0.64 (0.38-1.07)	.09	0.65 (0.36-1.15)	.10	0.77 (0.43-1.38)	.40
Distance to clinic	1.04 (1.02-1.07)	.001	1.04 (1.02-1.06)	.001	1.05 (1.04-1.07)	<.001	1.05 (1.03-1.06)	<.001
**Multigravid**								
PTD	1.74 (1.09-2.76)	.02	1.76 (1.05-2.95)	.03	1.57 (1.15-2.15)	.005	1.62 (1.16-2.27)	.005
Maternal *P falciparum*	1.46 (1.23-1.73)	<.001	1.54 (1.27-1.86)	<.001	1.37 (1.17-1.61)	<.001	1.37 (1.16-1.61)	<.001
ITN use	0.74 (0.63-0.87)	<.001	0.79 (0.66-0.95)	.01	0.79 (0.69-0.91)	.001	0.89 (0.77-1.03)	.10
Hemoglobin AC level	1.15 (0.89-1.49)	.30	0.97 (0.71-1.31)	.80	1.01 (0.82-1.24)	.90	1.07 (0.86-1.33)	.50
Hemoglobin AS level	0.91 (0.72-1.17)	.50	0.86 (0.66-1.11)	.20	0.93 (0.73-1.19)	.60	0.92 (0.71-1.19)	.50
Distance to clinic	1.04 (1.02-1.06)	<.001	1.04 (1.02-1.05)	<.001	1.04 (1.04-1.05)	<.001	1.04 (1.03-1.05)	<.001

Abbreviations: PTD, preterm delivery; HR, hazard ratio; IRR, incidence rate ratio; ITN, insecticide-treated net; NA, not applicable.

^a^ Models were adjusted for ITN use, distance to clinic, hemoglobin type, and calendar day of birth. Model that included all women was also adjusted for gravidity.

We then evaluated the association between PTD and the rate of infection in the offspring. The incidence rates (IRs) of infection per person-years among offspring born preterm were 0.63 (95% CI, 0.40-0.99) among primigravid, 1.01 (95% CI, 0.65-1.57) among secundigravid, and 1.65 (95% CI, 1.23-2.22) among multigravid women. IRs among offspring born at full term were 0.90 (95% CI, 0.79-1.03) among primigravid, 0.92 (95% CI, 0.80-1.06) among secundigravid, and 1.02 (95% CI, 0.95-1.09) among multigravid women. Negative binomial models were fitted to evaluate the association between PTD and the IR of *P falciparum* infections. In the model adjusted for multiple covariates including gravidity, the infection rate was 1.41 (95% CI, 1.24-1.61) times higher in offspring of PM-positive mothers, and PTD did not increase the IR (Table 2).

To evaluate whether PTD modified the IR differently in offspring of primigravid and multigravid women, the IR ratio (IRR) was estimated in gravidity-stratified models. Unlike PM, PTD was associated with a higher IR in multigravid offspring (adjusted IRR, 1.62; 95% CI, 1.16-2.27) but not in offspring of primigravid and secundigravid women (Table 2).

We further evaluated the association between PTD and the hazard of first parasitemia or the IR of parasitemia within subgroups defined by both gravidity and PM status based on the results of a Cox proportional hazards model and negative binomial model with an interaction term among PTD, PM, and gravidity. For children of secundigravid and multigravid women who were not infected during pregnancy, the HRs for first parasitemia in children born preterm were 2.17 (95% CI, 1.25-3.75; *P* = .006) and 3.36 (95% CI, 1.90-5.93; *P* < .001), respectively, compared with full-term children (Table 3). The adjusted IRR for preterm children of uninfected multigravid women was 2.74 (95% CI, 1.80-4.18) times the IRR for full-term children (Table 3). In models adjusted for growth rate and educational level, PTD remained independently associated with a higher hazard of first infection and higher IR of *P falciparum* infections in offspring of PM-negative multigravidae (eTable 4 in Supplement 1).

**Table 3.** HR for Time to First Parasitemia and IRR of Parasitemia Among Children Born Preterm vs Term for Strata of Maternal Infection and Gravidity^a^

Term	Adjusted HR (95% CI)	*P* value	Adjusted IRR (95% CI)	*P* value
PTD: PM-positive primigravid	0.67 (0.40-1.12)	.10	0.79 (0.46-1.34)	.40
PTD: PM-positive secundigravid	1.07 (0.60-1.90)	.80	0.84 (0.45-1.54)	.60
PTD: PM-positive multigravid	1.35 (0.69-2.63)	.40	1.08 (0.70-1.65)	.70
PTD: PM-negative primigravid	0.88 (0.30-2.55)	.80	0.52 (0.22-1.25)	.10
PTD: PM-negative secundigravid	2.17 (1.25-3.75)	.006	1.34 (0.69-2.63)	.40
PTD: PM-negative multigravid	3.36 (1.90-5.93)	<.001	2.74 (1.80-4.18)	<.001
ITN use	0.82 (0.71-0.93)	.003	0.94 (0.84-1.05)	.30
Hemoglobin AC	0.88 (0.68-1.13)	.30	0.96 (0.80-1.15)	.70
Hemoglobin AS	0.77 (0.62-0.95)	.02	0.86 (0.69-1.06)	.10
Distance to clinic	1.04 (1.03-1.05)	<.001	1.04 (1.04-1.05)	<.001

Abbreviations: HR, hazard ratio; IRR, incidence rate ratio; ITN, insecticide-treated net; PM, pregnancy malaria; PTD, preterm delivery.

^a^ Models were adjusted for ITN use, distance to clinic, hemoglobin type, and calendar day of birth.

### Association of PTD With Susceptibility to Malaria Disease

We examined whether PTD was associated with the hazard of first clinical malaria, IR of clinical malaria, and IR of severe malaria. In the Cox proportional hazards regression model, the AHR of first clinical malaria was significantly greater for children born to PM-positive vs PM-negative primigravid (2.40; 95% CI, 1.59-3.63), secundigravid (1.52; 95% CI, 1.03-2.25), and multigravid (1.43; 95% CI, 1.17-1.75) women (eTable 5 in Supplement 1). PM also increased the IR of clinical malaria in all offspring; the adjusted IR of clinical malaria in offspring of PM-positive primigravid women was 2.20 (95% CI, 1.50-3.23) times that of offspring of uninfected mothers; PM-positive secundigravid women, 1.55 (95% CI ,1.09-2.19); and PM-positive multigravid women, 1.33 (95% CI, 1.12-1.58) (eTable 6 in Supplement 1). However, PTD was not associated with a significant increase in the hazard of first clinical malaria or the IR of clinical malaria (eTables 5 and 6 in Supplement 1). Similarly, PTD did not significantly increase the incidence rate of severe malaria. There was not a significant association between PM and the IR of severe malaria (IRR, 1.43; 95% CI, 0.94-2.16; *P* = .09) (eTable 7 in Supplement 1). In this cohort, 7 of 124 children who experienced severe malaria were born preterm; whether PTD increased the IR of severe malaria should be further assessed with a larger sample.

## Discussion

Pregnant women and young children are most susceptible to *P falciparum* infection. PTD is one of the adverse outcomes associated with PM. In this cohort of mother-child pairs, PM was associated with a significantly increased risk of PTD in nonimmune primigravid women.^9^ In the offspring who were followed up longitudinally, PM was associated with higher hazards of *P falciparum* infection and clinical malaria and higher IR of *P falciparum* infection and clinical malaria in all offspring. We found that PTD was independently associated with a higher hazard of first *P falciparum* infection and IR of infection in the offspring of PM-negative multigravid women. We speculate that because PM alone is a strong risk modifier of child susceptibility to infection, PTD did not further increase the risk in children exposed in utero to malarial antigens.

PTD has been reported as a risk factor for infections other than malaria. Previous studies reported PTD as one of the risk factors for increased hospitalization rate due to respiratory syncytial virus infection in the first 1 to 2 years of life.^20,21^ A large study in Norway^22^ evaluated the association of PTD with the risk of hospitalization due to infectious disease from birth to age 50 years. Among children aged 1 to 5 years, the relative risk of hospitalization among late (gestational age, 34-36 weeks) preterm offspring was 1.7 (95% CI, 1.6-1.8). The impact of PTD, although smaller, was also observed among adults aged 30 to 50 years.^22^ Data collected in 6 countries (INTERBIO-21st study) reported that at the age of 2 years, PTD alone increased the odds of contracting an infection (OR, 1.4; 95% CI, 1.2-1.6).^23^ The question remained as to why PTD was associated with higher hazards of first infection in PM-negative secundigravid and multigravid but not primigravid women, and a higher IR only in offspring of PM-negative multigravid women (Table 3), similar to that reported for other infections. Several potential confounders, such as distance between home and the clinic, educational attainment, and growth rate, that have been previously associated with malaria risks were included in the analysis.^24,25^ Although we observed similar associations with malaria infection as previously described, PTD remained an independent factor associated with a higher HR in offspring of PM-negative secundigravid and multigravid women and a higher IR of *P falciparum* in preterm children of PM-negative multigravid women.

Alternatively, we hypothesize that offspring of primigravid and multigravid women may differ in their T-cell profiles. Previous studies described that immune cell profile varies at birth between preterm and full-term newborns.^26^ By the age of 3 months, the frequency of immune cells, such as neutrophils, naïve CD4^+^ T cells, dendritic cells, and B cells, was similar between full-term and preterm infants.^27,28^ Transcriptomic analysis of immune cells showed some differences, such as downregulation of genes associated with interferon-γ production and T cell proliferation, remained at 3 months of age, which may explain differences in susceptibility to infections between full-term and preterm children.^27^ In the context of this study, further studies comparing immune cell phenotypes and acquisition of immunity to malaria between full-term and preterm offspring of *P falciparum*–infected and –uninfected primigravid and multigravid women may explain the gravidity-dependent association between PTD and malaria during early life.

### Limitations

Our study has several limitations. Although the cohort included 1679 children, the sample size for PTD is small. Because PM is associated with a higher risk of PTD in primigravid women and a higher susceptibility to malaria in the offspring, models were stratified by these covariates, resulting in a smaller sample size per group. Another limitation is that most women enrolled during the second trimester, and infections that may have occurred prior to enrollment were unaccounted for, possibly resulting in misclassification of some of uninfected women. Primigravid women are infected with higher parasite densities than multigravid women, and higher parasite densities during preenrollment infections may modify the interaction among gravidity, PM, and PTD differently. The sample size of truly uninfected primigravid women may not allow observation of the impact of PTD on the child’s susceptibility to *P falciparum* infection. To avoid excluding children with missing hemoglobin type from the analysis, missing hemoglobin types were imputed with a single imputation based on the proportion of nonmissing hemoglobin types. This missingness and imputation may lead to some misclassification of hemoglobin type, potentially affecting the association between hemoglobin type and outcomes. It is also possible that singly imputing this variable may lead to some underestimation of variance, particularly for this association. However, child hemoglobin type is not expected to affect PTD, and so this imputation would not be expected to contribute to inadequate confounder adjustment. In addition, models were adjusted for multiple factors associated with malaria risks. However, we cannot rule out that there are other potential risk factors that were not accounted for.

## Conclusions

In this cohort study of children followed up from birth through early childhood, maternal malaria infection during pregnancy was associated with increased child susceptibility to malaria during the first years of life based on higher hazards of first infection and first clinical malaria and higher IRs of *P falciparum* infection and clinical malaria. These changes were observed in all exposed offspring regardless of maternal gravidity. However, PTD was associated with higher child susceptibility to *P falciparum* infection only among offspring of uninfected multigravid women. The parity-dependent response to PTD remains to be further explored in a larger study sample with monitoring for immune cell profiles.

## Notes

Supplement 1. **eTable 1.** Study Population Stratified by Gravidity and Maternal Infection During Pregnancy **eTable 2.** Unadjusted and Adjusted Hazard Ratios for First Parasitemia **eTable 3.** Unadjusted and adjusted Odds Ratios and Relative Risks of Any Parasitemia Longitudinally During Early Childhood Associated With Maternal Malaria During Pregnancy, Stratified by Gravidity **eTable 4.** Adjusted Hazard Ratio for First Parasitemia and Incidence Rate Ratio (IRR) for Parasitemia During Infancy and Early Childhood Among Preterm and Term Newborns **eTable 5.** Unadjusted and Adjusted Hazard Ratio for First Clinical Malaria During Infancy and Early Childhood Among Preterm and Term Newborns **eTable 6.** Unadjusted and Adjusted Incidence Rate Ratio (IRR) for Clinical Malaria During Infancy and Early Childhood Among Preterm and Term Newborns **eTable 7.** Unadjusted and Adjusted Incidence Rate Ratio (IRR) for Severe Malaria During Infancy and Early Childhood Among Preterm and Term Newborns **eFigure.** Kaplan-Meier Estimate of Percent Without Parasitemia Over Time by Maternal Infection History

Supplement 2.
Data Sharing Statement
