# Supplementary material for: Preterm Birth and Malaria Susceptibility in Offspring of Uninfected Multigravid Women
Source: JAMA Netw Open. 2025 Sep 16;8(9):e2532179. doi: 10.1001/jamanetworkopen.2025.32179 (PMC12441872; doi:10.1001/jamanetworkopen.2025.32179)
Supplement: Supplement 1. — eTable 1. Study Population Stratified by Gravidity and Maternal Infection During Pregnancy eTable 2. Unadjusted and Adjusted Hazard Ratios for First Parasitemia eTable 3. Unadjusted and adjusted Odds Ratios and Relative Risks of Any Parasitemia Longitudinally During Early Childhood Associated With Maternal Malaria During Pregnancy, Stratified by Gravidity eTable 4. Adjusted Hazard Ratio for First Parasitemia and Incidence Rate Ratio (IRR) for Parasitemia During Infancy and Early Childhood Among Preterm and Term Newborns eTable 5. Unadjusted and Adjusted Hazard Ratio for First Clinical Malaria During Infancy and Early Childhood Among Preterm and Term Newborns eTable 6. Unadjusted and Adjusted Incidence Rate Ratio (IRR) for Clinical Malaria During Infancy and Early Childhood Among Preterm and Term Newborns eTable 7. Unadjusted and Adjusted Incidence Rate Ratio (IRR) for Severe Malaria During Infancy and Early Childhood Among Preterm and Term Newborns eFigure. Kaplan-Meier Estimate of Percent Without Parasitemia Over Time by Maternal Infection History [file jamanetwopen-e2532179-s001.pdf]

Supplementary Online Content

Barry A, Dang L, Sibide Y, et al. Preterm birth and increased malaria susceptibility in offspring of uninfected multigravid women. *JAMA Netw Open.* 2025;8(9):e2532179. doi:10.1001/jamanetworkopen.2025.32179

eTable 1. Study Population Stratified by Gravidity and Maternal Infection During Pregnancy
eTable 2. Unadjusted and Adjusted Hazard Ratios for First Parasitemia
eTable 3. Unadjusted and adjusted Odds Ratios and Relative Risks of Any Parasitemia Longitudinally During Early Childhood Associated With Maternal Malaria During Pregnancy, Stratified by Gravidity
eTable 4. Adjusted Hazard Ratio for First Parasitemia and Incidence Rate Ratio (IRR) for Parasitemia During Infancy and Early Childhood Among Preterm and Term Newborns
eTable 5. Unadjusted and Adjusted Hazard Ratio for First Clinical Malaria During Infancy and Early Childhood Among Preterm and Term Newborns
eTable 6. Unadjusted and Adjusted Incidence Rate Ratio (IRR) for Clinical Malaria During Infancy and Early Childhood Among Preterm and Term Newborns
eTable 7. Unadjusted and Adjusted Incidence Rate Ratio (IRR) for Severe Malaria During Infancy and Early Childhood Among Preterm and Term Newborns
eFigure. Kaplan-Meier Estimate of Percent Without Parasitemia Over Time by Maternal Infection History

This supplementary material has been provided by the authors to give readers additional information about their work.

© 2025 Barry A et al. *JAMA Network Open*.

eTable 1. Study Population Stratified by Gravidity and Maternal Infection During Pregnancy

Group	n	Gestational age at enrollment (weeks) Mean (SD)[1]	[2]Birth during transmission season n (%)	Child follow-up duration (months) Mean (SD)	Distance (km) Mean (SD)	Hb AC (%)[4]	Hb AS (%)[1]	[5]ITN (%)[1]	Child Severe malaria during follow-up n (%)
Primigravid, PM+	278	20.92 (6.51)	136 (48.92)	21.17 (15.58)	3.16 (4.96)[1]	10.79	7.91	38.49	24 (8.63)[1]
Primigravid, PM-	104	21.39 (7.89)	46 (44.23)	22.06 (14.50)	2.26 (5.88)	11.54	7.96	48.08	4 (3.85)
Secundigravid, PM+	215	20.65 (6.56)	104 (48.37)	21.42 (15.53)	4.12 (6.54)[1]	10.23	11.16	53.49	21 (9.77)[3]
Secundigravid, PM-	109	20.71 (7.51)	49 (44.95)	20.77 (14.04)	2.60 (5.00)	11.01	11.01	53.21	2 (1.83)
Multigravid, PM+	635	20.65 (6.96)	315 (49.60)	24.09 (15.70)	5.28 (7.76)[3]	8.19	9.76	56.22	48 (7.56)[1]
Multigravid, PM-	346	21.01 (6.75)	115 (33.24)	26.03 (16.07)	3.65 (7.47)	12.72	9.25	53.76	25 (7.23)

Abbreviations: ITN, insecticide-treated net; HGB, hemoglobin.
[1] No significant differences in the comparison of PM+ to PM-
[2] Peak malaria transmission in Ouélessébougou, Mali occurs during the rainy season from July to December
[3] $p<0.05$ in the comparison of PM+ to PM-
[4] Proportions were different ($p<0.05$) only for the comparison between PM- and PM+ multigravidae
[5] Maternal insecticide-treated net (ITN) use

© 2025 Barry A et al. *JAMA Network Open*.

eTable 2. Unadjusted and Adjusted Hazard Ratios for First Parasitemia

Term	Unadjusted HR (95% CI)	P value	Adjusted HR (95% CI)	P value
**All**				
Maternal Pf	1.50 (1.31-1.73)	<0.0001	1.56 (1.34-1.82)	<0.0001
Primigravid	0.87 (0.75-1.02)	0.08	0.89(0.76-1.04)	0.1
Secundigravid	1.01 (0.86-1.19)	0.9	1.03 (0.87-1.22)	0.8
Multigravid	Ref			
ITN use	0.79 (0.69-0.89)	<0.0001	0.81 (0.70-0.93)	0.002
HGB AC	1.01 (0.82-1.26)	0.9	0.89 (0.70-1.15)	0.4
HGB AS	0.81 (0.66-0.99)	0.049	0.79 (0.64-0.98)	0.03
Distance	1.04 (1.03-1.06)	<0.0001	1.04 (1.03-1.05)	<0.0001
**Primigravid**				
Maternal Pf	1.95 (1.41-2.69)	<0.0001	1.86 (1.31-2.63)	<0.0001
ITN use	0.80 (0.61-1.06)	0.1	0.92 (0.68-1.23)	0.6
HGB AC	1.06 (0.68-1.65)	0.8	0.97 (0.59-1.60)	0.9
HGB AS	0.69 (0.38-1.27)	0.2	0.70 (0.40-1.24)	0.2
Distance	1.06 (1.03-1.10)	<0.0001	1.06 (1.02-1.09)	<0.0001
**Secundigravid**				
Maternal Pf	1.35 (0.98-1.85)	0.07	1.40 (1.00-1.96)	0.049
ITN use	0.88 (0.66-1.18)	0.4	0.81 (0.60-1.11)	0.2
HGB AC	0.54 (0.28-1.04)	0.07	0.52 (0.28-0.98)	0.04
HGB AS	0.57 (0.33-0.96)	0.04	0.64 (0.38-1.08)	0.09
Distance	1.04 (1.02-1.07)	0.001	1.04 (1.02-1.07)	0.001
**Multigravid**				
Maternal Pf	1.46 (1.23-1.73)	<0.0001	1.54 (1.27-1.87)	<0.0001
ITN use	0.74 (0.63-0.87)	<0.0001	0.79 (0.66-0.95)	0.01
HGB AC	1.15 (0.89-1.49)	0.3	0.99 (0.72-1.34)	0.9
HGB AS	0.91 (0.72-1.17)	0.5	0.86 (0.66-1.11)	0.2
Distance	1.04 (1.02-1.06)	<0.0001	1.04 (1.02-1.05)	<0.0001

Abbreviations: HR, hazard ratio; CI, confidence interval; ITN, insecticide-treated net; HGB, hemoglobin. Adjusted HR: model was adjusted for ITN, distance, hemoglobin type, and calendar day of birth. Model that included all women (All) was also adjusted for gravidity.

© 2025 Barry A et al. *JAMA Network Open*.

eTable 3. Unadjusted and adjusted Odds Ratios and Relative Risks of Any Parasitemia Longitudinally During Early Childhood Associated With Maternal Malaria During Pregnancy, Stratified by Gravidity

Term	Unadjusted OR (95% CI)	Adjusted OR (95% CI)	Unadjusted RR (95% CI)	Adjusted RR (95% CI)
PM- primigravidae	0.63 (0.43-0.93)	0.75 (0.52-1.09)	0.65 (0.45-0.94)	0.78 (0.54-1.10)
PM- secundigravidae	0.89 (0.61-1.30)	1.04 (0.73-1.49)	0.90 (0.63-1.27)	1.04 (0.74-1.43)
PM- multigravidae	Reference		Reference	
PM+ primigravidae	1.30 (1.04-1.61)	1.48 (1.19-1.85)	1.27 (1.04-1.54)	1.43 (1.17-1.74)
PM+ secundigravidae	1.28 (1.00-1.64)	1.42 (1.10-1.83)	1.25 (1.00-1.57)	1.37 (1.09-1.72)
PM+ multigravidae	1.46 (1.21-1.76)	1.51 (1.24-1.83)	1.41 (1.19-1.66)	1.45 (1.22-1.73)

Abbreviations: OR, odd ratio; RR, relative risk; CI, confidence interval.
Adjusted OR: model was adjusted for ITN, age, transmission season at time of bloodsmear and day of birth as a cubic spline, distance from the clinic. A quadratic term for age was included for comparability with a previous study (Mutabingwa TK, Bolla MC, Li JL, Domingo GJ, Li X, Fried M, et al. Maternal malaria and gravidity interact to modify infant susceptibility to malaria. *PLoS Med.* 2005;2(12):e407). Odds ratio estimates were converted to relative risks via the method of substitution.

© 2025 Barry A et al. *JAMA Network Open*.

eTable 4. Adjusted Hazard Ratio for First Parasitemia and Incidence Rate Ratio (IRR) for Parasitemia During Infancy and Early Childhood Among Preterm and Term Newborns

Term	Adjusted HR (95% CI)	P value	Adjusted IRR (95% CI)	P value
**Primigravid**				
PTD	0.81 (0.46-1.44)	0.5	0.91 (0.59-1.41)	0.7
Maternal Pf	1.67 (1.14-2.43)	0.008	1.78 (1.29-2.45)	<0.0001
ITN use	1.00 (0.72-1.39)	0.9	1.06 (0.82-1.38)	0.6
HGB AC	0.96 (0.52-1.77)	0.9	1.07 (0.69-1.66)	0.8
HGB AS	0.72 (0.39-1.35)	0.3	0.80 (0.51-1.27)	0.3
Distance	1.06 (1.02-1.09)	0.002	1.06 (1.04-1.09)	<0.0001
Primary school[a]	0.61 (0.40-0.95)	0.03	0.75 (0.52-1.10)	0.1
Secondary school[a]	0.46 (0.28-0.76)	0.002	0.71 (0.47-1.06)	0.1
Growth rate	0.42 (0.04-4.47)	0.5	0.53 (0.09-3.20)	0.5
**Secundigravid**				
PTD	1.27 (0.75-2-14)	0.4	0.94 (0.54-1.62)	0.8
Maternal Pf	0.99 (0.67-1.45)	0.9	1.07 (0.78-1.47)	0.7
ITN use	0.94 (0.65-1.35)	0.7	1.06 (0.79-1.41)	0.7
HGB AC	0.27 (0.10-0.77)	0.01	0.30 (0.15-0.63)	0.002
HGB AS	0.70 (0.37-1.30)	0.3	0.86 (0.51-1.45)	0.6
Distance	1.04 (1.01-1.06)	0.001	1.04 (1.02-1.07)	<0.0001
Primary school[a]	0.91 (0.61-1.37)	0.6	0.84 (0.60-1.18)	0.3
Secondary school[a]	0.45 (0.27-0.74)	0.002	0.51 (0.34-0.75)	0.001
Growth rate	0.18 (0.01-4.28)	0.3	0.59 (0.06-6.11)	0.7
**Multigravid**				
PTD	1.84 (1.04-3.26)	0.04	1.72 (1.14-2.61)	0.01
Maternal Pf	1.45 (1.18-1.78)	<0.0001	1.37 (1.16-1.62)	<0.0001
ITN use	0.77 (0.64-0.94)	0.009	0.88 (0.75-1.03)	0.1
HGB AC	0.89 (0.63-1.27)	0.5	1.14 (0.88-1.46)	0.3
HGB AS	1.00 (0.76-1.31)	0.9	0.98 (0.76-1.26)	0.9
Distance	1.04 (1.02-1.05)	<0.0001	1.04 (1.03-1.05)	<0.0001
Primary school[a]	0.78 (0.64-0.95)	0.01	0.75 (0.64-0.88)	<0.0001
Secondary school[a]	0.46 (0.33-0.66)	<0.0001	0.50 (0.38-0.67)	<0.0001
Growth rate	1.97 (0.53-7.25)	0.3	2.52 (0.73-8.68)	0.1

Abbreviations: PTD, preterm delivery; ITN, insecticide-treated net; HGB, hemoglobin.
[a]No education was used as reference.
Weight-for-height was calculated as weight divided by height. Growth rate was defined as the rate of change in weight-for-height during the first 3 months of life.

© 2025 Barry A et al. *JAMA Network Open*.

eTable 5. Unadjusted and Adjusted Hazard Ratio for First Clinical Malaria During Infancy and Early Childhood Among Preterm and Term Newborns

Term	Unadjusted HR (95% CI)	P value	Adjusted HR (95% CI)	P value
**All**				
PTD	1.12 (0.82-1.53)	0.5	1.13 (0.80-1.59)	0.5
Maternal Pf	1.56 (1.34-1.82)	<0.0001	1.59 (1.35-1.87)	<0.0001
Primigravid	0.88 (0.74-1.04)	0.1	0.89 (0.74-1.06)	0.2
Secundigravid	0.98 (0.81-1.18)	0.8	0.97 (0.80-1.18)	0.8
Multigravid	Ref			
ITN use	0.89 (0.78-1.02)	0.1	0.92 (0.80-1.07)	0.3
HGB AC	0.91 (0.72-1.15)	0.4	0.83 (0.65-1.08)	0.2
HGB AS	0.62 (0.48-0.80)	<0.0001	0.59 (0.46-0.76)	<0.0001
Distance	1.04 (1.02-1.05)	<0.0001	1.04 (1.02-1.05)	<0.0001
**Primigravid**				
PTD	0.91 (0.55-1.52)	0.7	0.9 (0.53-1.55)	0.7
Maternal Pf	2.53 (1.72-3.72)	<0.0001	2.40 (1.59-3.63)	<0.0001
ITN use	0.75 (0.55-1.02)	0.07	0.83 (0.60-1.15)	0.3
HGB AC	1.19 (0.76-1.86)	0.5	1.13 (0.67-1.93)	0.6
HGB AS	0.46 (0.21-0.98)	0.045	0.46 (0.21-1.00)	0.05
Distance	1.06 (1.02-1.09)	0.001	1.04 (1.01-1.08)	0.008
**Secundigravid**				
PTD	1.30 (0.72-2.36)	0.4	1.08 (0.55-2.12)	0.8
Maternal Pf	1.53 (1.05-2.21)	0.03	1.52 (1.03-2.25)	0.04
ITN use	1.09 (0.78-1.51)	0.6	1.03 (0.73-1.44)	0.9
HGB AC	0.42 (0.21-0.84)	0.01	0.42 (0.22-0.81)	0.01
HGB AS	0.49 (0.27-0.87)	0.02	0.54 (0.30-0.98)	0.04
Distance	1.03 (1.01-1.05)	0.004	1.03 (1.004-1.05)	0.02
**Multigravid**				
PTD	1.49 (0.85-2.59)	0.2	1.54 (0.84-2.82)	0.2
Maternal Pf	1.40 (1.16-1.68)	<0.0001	1.43 (1.17-1.75)	0.001
ITN use	0.88 (0.74-1.05)	0.1	0.95 (0.78-1.15)	0.6
HGB AC	0.99 (0.74-1.34)	0.9	0.87 (0.62-1.23)	0.4
HGB AS	0.70 (0.53-0.94)	0.02	0.63 (0.47-0.86)	0.003
Distance	1.04 (1.02-1.05)	<0.0001	1.04 (1.02-1.05)	<0.0001

Abbreviations: HR, hazard ratio; CI, confidence interval; PTD, preterm delivery; ITN, insecticide-treated net; HGB, hemoglobin.
Adjusted HR: model was adjusted for ITN, distance, hemoglobin type, and calendar day of birth. Model that included all women (All) was also adjusted for gravidity.

© 2025 Barry A et al. *JAMA Network Open*.

eTable 6. Unadjusted and Adjusted Incidence Rate Ratio (IRR) for Clinical Malaria During Infancy and Early Childhood Among Preterm and Term Newborns

Term	Unadjusted IRR (95% CI)	P value	Adjusted IRR (95% CI)	P value
**All**				
PTD	1.05 (0.76-1.41)	0.7	1.10 (0.82-1.48)	0.5
Maternal Pf	1.47 (1.28-1.70)	<0.0001	1.49 (1.29-1.71)	<0.0001
Primigravid	0.88 (0.75-1.03)	0.1	0.88 (0.75-1.04)	0.1
Secundigravid	0.93 (0.79-1.10)	0.4	0.91 (0.77-1.07)	0.3
Multigravid	Ref			
ITN use	0.95 (0.84-1.08)	0.4	1.00 (0.88-1.14)	0.9
HGB AC	0.85 (0.68-1.05)	0.1	0.87 (0.69-1.08)	0.2
HGB AS	0.60 (0.46-0.77)	<0.0001	0.58 (0.45-0.74)	<0.0001
Distance	1.04 (1.03-1.04)	<0.0001	1.03 (1.02-1.04)	<0.0001
**Primigravid**				
PTD	0.77 (0.49-1.21)	0.3	0.74 (0.47-1.16)	0.2
Maternal Pf	2.40 (1.66-3.47)	<0.0001	2.20 (1.50-3.23)	<0.0001
ITN use	0.91 (0.68-1.22)	0.5	1.05 (0.80-1.37)	0.7
HGB AC	0.67 (0.47-0.97)	0.04	0.71 (0.46-1.10)	0.1
HGB AS	0.33 (0.16-0.67)	0.002	0.33 (0.16-0.65)	0.001
Distance	1.05 (1.03-1.08)	<0.0001	1.04 (1.01-1.07)	0.004
**Secundigravid**				
PTD	1.01 (0.60-1.70)	0.9	1.02 (0.55-1.89)	0.9
Maternal Pf	1.61 (1.14-2.28)	0.007	1.55 (1.09-2.19)	0.02
ITN use	0.99 (0.73-1.34)	0.9	0.95 (0.71-1.27)	0.7
HGB AC	0.39 (0.21-0.73)	0.003	0.39 (0.20-0.73)	0.004
HGB AS	0.46 (0.24-0.85)	0.01	0.53 (0.28-1.02)	0.06
Distance	1.04 (1.02-1.06)	<0.0001	1.03 (1.01-1.05)	0.006
**Multigravid**				
PTD	1.55 (0.98-2.45)	0.06	1.55 (0.98-2.44)	0.06
Maternal Pf	1.32 (1.11-1.58)	0.002	1.33 (1.12-1.58)	0.001
ITN use	0.93 (0.80-1.09)	0.4	1.02 (0.87-1.20)	0.8
HGB AC	1.00 (0.78-1.30)	0.9	1.06 (0.80-1.39)	0.7
HGB AS	0.70 (0.52-0.95)	0.02	0.65 (0.49-0.87)	0.004
Distance	1.03 (1.02-1.04)	<0.0001	1.03 (1.02-1.04)	<0.0001

Abbreviations: HR, hazard ratio; CI, confidence interval; PTD, preterm delivery; ITN, insecticide-treated net; HGB, hemoglobin.
Adjusted HR: model was adjusted for ITN, distance, hemoglobin type, and calendar day of birth. Model that included all women (All) was also adjusted for gravidity.

© 2025 Barry A et al. *JAMA Network Open*.

eTable 7. Unadjusted and Adjusted Incidence Rate Ratio (IRR) for Severe Malaria During Infancy and Early Childhood Among Preterm and Term Newborns

Term	Unadjusted IRR (95% CI)	P value	Adjusted IRR (95% CI)	P value
**All**				
PTD	1.22 (0.56-2.66)	0.6	1.06 (0.47-2.39)	0.9
Maternal Pf	1.61 (1.08-2.41)	0.02	1.43 (0.94-2.16)	0.09
Primigravid	1.16 (0.76-1.79)	0.5	1.16 (0.73-1.85)	0.5
Secundigravid	1.13 (0.71-1.79)	0.6	1.16 (0.73-1.84)	0.5
Multigravid	Ref			
ITN use	0.53 (0.37-0.76)	<0.0001	0.62 (0.43-0.91)	0.01
HGB AC	0.50 (0.23-1.05)	0.07	0.46 (0.21-1.03)	0.06
HGB AS	0.27 (0.10-0.75)	0.01	0.26 (0.10-0.72)	0.009
Distance	1.06 (1.04-1.08)	<0.0001	1.05 (1.04-1.07)	<0.0001

Abbreviations: IRR, incidence rate ratio; CI, confidence interval; PTD, preterm delivery; ITN, insecticide-treated net; HGB, hemoglobin. Adjusted IRR: model was adjusted for gravidity, ITN, distance, hemoglobin type, and calendar day of birth.

© 2025 Barry A et al. *JAMA Network Open*.

eFigure. Kaplan-Meier Estimate of Percent Without Parasitemia Over Time by Maternal Infection History Unadjusted age to first malaria infection in the whole cohort (A), offsprings of primigravidae (B), offsprings of secundigravidae (C) and offsprings of multigravidae (D). In adjusted Cox proportional hazards models (eTable 2 in Supplement), maternal infection was associated with a significantly higher hazard of first parasitemia in the offsprings of all women regardless of gravidity

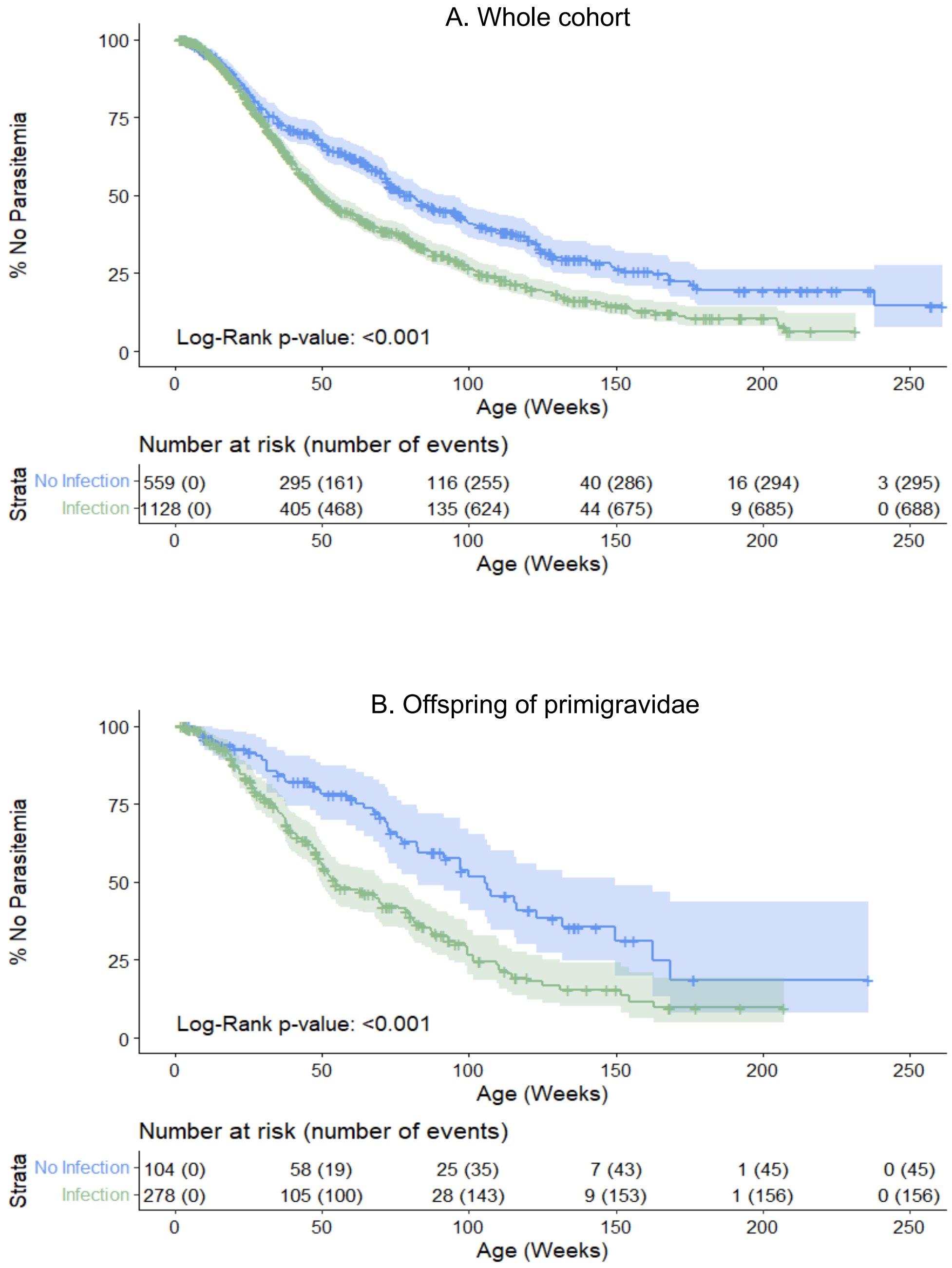

© 2025 Barry A et al. *JAMA Network Open*.

C. Offspring of secundigravidae

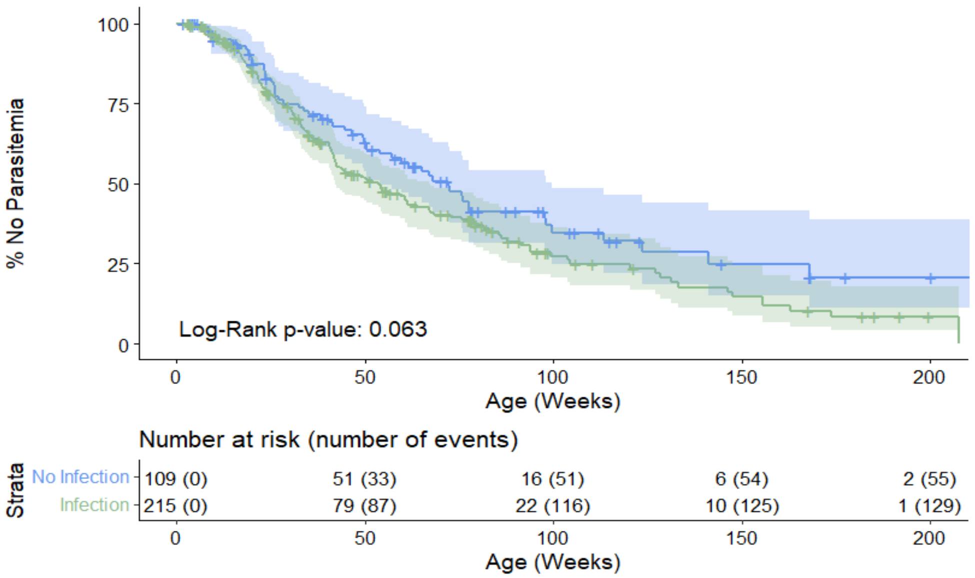

D. Offspring of multigravidae

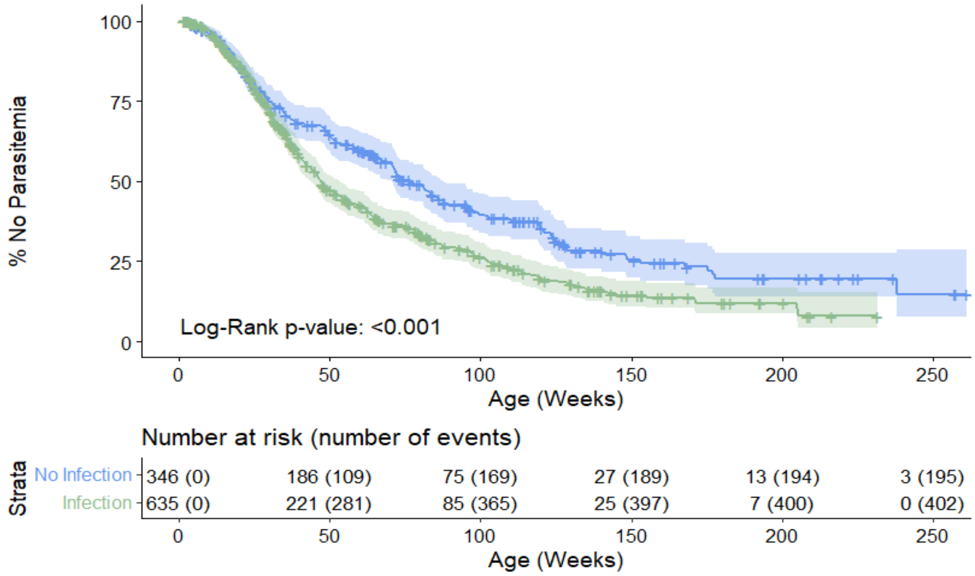

© 2025 Barry A et al. *JAMA Network Open*.
