# Supplementary material for: Preterm Birth and Malaria Susceptibility in Offspring of Uninfected Multigravid Women
Source: JAMA Netw Open. 2025 Sep 16;8(9):e2532179. doi: 10.1001/jamanetworkopen.2025.32179 (PMC12441872; doi:10.1001/jamanetworkopen.2025.32179)
Supplement: Supplement 2. — Data Sharing Statement [file jamanetwopen-e2532179-s002.pdf]

Data Sharing Statement

Barry. Preterm Birth and Malaria Susceptibility in Offspring of Uninfected Multigravid Women. *JAMA Netw Open*. Published September 16, 2025. doi:10.1001/jamanetworkopen.2025.32179

Data
Data available: Yes
Data types: Deidentified participant data
How to access data: Request for data must be sent to Michal Fried, email: michal.fried@nih.gov
When available: With publication

Supporting Documents
Document types: None

Additional Information
Who can access the data: Data will be available for researchers whose proposed use of the data has been approved.
Types of analyses: Data will be made available for meta-analysis
Mechanisms of data availability: Data will be made available after a signed data access agreement.
